# Compositional Shift of Oral Microbiota Following Surgical Resection of Tongue Cancer

**DOI:** 10.3389/fcimb.2020.600884

**Published:** 2020-11-23

**Authors:** Shinya Kageyama, Yuka Nagao, Jiale Ma, Mikari Asakawa, Ryoji Yoshida, Toru Takeshita, Akiyuki Hirosue, Yoshihisa Yamashita, Hideki Nakayama

**Affiliations:** ^1^ Section of Preventive and Public Health Dentistry, Division of Oral Health, Growth and Development, Faculty of Dental Science, Kyushu University, Fukuoka, Japan; ^2^ Department of Oral and Maxillofacial Surgery, Faculty of Life Sciences, Kumamoto University, Kumamoto, Japan; ^3^ OBT Research Center, Faculty of Dental Science, Kyushu University, Fukuoka, Japan

**Keywords:** saliva, microbiome, 16S ribosomal RNA, next-generation sequencing, quantitative real-time PCR, tongue neoplasms, oral surgery, glossectomy

## Abstract

Salivary microbiota is considered a source of microorganisms for the respiratory and digestive tracts, and a trigger for diseases in these distant organs. Meanwhile, the microbiota on the tongue surface is thought to be a major source of salivary microbiota. Therefore, surgical resection of the tongue for definitive treatment of oral cancer could drastically change the salivary bacterial balance and virulence. Here, we investigated the shift of the salivary microbiota following surgical resection in patients with tongue cancer. The stimulated saliva samples were collected from 25 tongue cancer patients pre- and post-resection of the tongue, and bacterial density and composition was determined using quantitative PCR analysis and 16S ribosomal RNA (rRNA) gene sequencing, respectively. Although no significant difference in the total bacterial density in saliva pre- and post-surgery was observed, the bacterial composition significantly differed according to the analysis of similarity. Among predominant operational taxonomic units (OTUs) with ≥1% of relative abundance, the proportions of OTUs corresponding to *Streptococcus salivarius*, *Prevotella* *melaninogenica*, and *Prevotella* *histicola* were significantly decreased following the tongue resection. On the other hand, the proportions of OTUs corresponding to *Lautropia* *mirabilis*, *Neisseria* *flava*, *Streptococcus* *sanguinis*, and *Fusobacterium nucleatum*, known to be inhabitants of dental plaque, were significantly increased. These results suggest that surgical resection of the tongue causes a compositional shift of the salivary microbiota, characterized by an increase in bacterial species derived from dental plaque, including periodontal pathogens. These results suggest the necessity of more careful and frequent postoperative oral care after surgical resection of tongue cancer.

## Introduction

Saliva is secreted from the salivary glands into the oral cavity and contains various oral debris including desquamated epithelial cells, food residue, and dense oral bacteria. Since, saliva is swallowed constantly, the salivary microbiota is considered as a source of microorganisms to respiratory and digestive tracts and a trigger of diseases in these distant organs. Recent studies suggested the association of oral bacteria with diseases occurring in organs far from oral cavity such as pneumonia, colorectal cancer, and inflammatory bowel disease (Gevers et al., 2014; Segal et al., 2016; Huffnagle et al., 2017; Kageyama et al., 2018; Kageyama et al., 2019; Schmidt et al., 2019; Yachida et al., 2019). The salivary microbiota is a mixture of bacteria shed from various oral niches, such as tongue dorsum, tooth surface, gingival crevice, and buccal mucosa. Among them, tongue microbiota is thought of a major source of salivary microbiota as the bacterial composition in saliva resembles that on tongue dorsum (Mager et al., 2003; Segata et al., 2012; Kageyama et al., 2017).

Cancer is a serious global health problem with a high mortality risk (Siegel et al., 2019). In Japan, the cancer-related deaths account for a quarter of all causes and are the leading causes of mortality for both sexes (Ministry of Health, Labour and Welfare, 2019). Oral cancer, predominantly oral squamous cell carcinoma, generally appears on the tongue, gingiva, floor of the mouth, and palate. Approximately 10,000 Japanese are diagnosed with oral cancer annually (Cancer Information Service, National Cancer Center Japan, 2020). Among them, tongue cancer is the most common malignancy and accounts for approximately 50% of the cases (Cancer Information Service, National Cancer Center Japan, 2020). A large number of epidemiological studies have demonstrated that lifestyle factors, such as smoking, alcohol intake, underweight, and low consumption of vegetables and fruits, are associated with oral cancer (Bosetti et al., 2000; Huang et al., 2003; Varela-Lema et al., 2010; Radoï et al., 2015). Regarding treatment, although internal radiotherapy is performed for T1–2 or superficial T3 cancers or concurrent chemoradiotherapy for advanced cancer, surgical resection of tumors is the most well-established definitive approach to oral cancer (Shah and Gil, 2009; Nibu et al., 2017; Colevas et al., 2018). Surgery reduces the area of the tongue surface with complex papillary structures, and the defect is reconstructed by foreign tissue in some cases. Therefore, the salivary bacterial balance and virulence could drastically change following surgical resection of tongue cancer.

In this study, we examined the salivary microbiota collected from patients with tongue cancer pre- and post-definitive surgery. We compared their bacterial density and composition using quantitative PCR analysis and 16S ribosomal RNA (16S rRNA) gene amplicon sequencing and confirmed how bacterial density and composition of salivary microbiota changed following tongue resection. This study aimed to characterize the bacterial shift before and after surgery and identify bacterial species that show drastic changes.

## Materials and Methods

### Study Subjects and Sample Collection

Study subjects of this study were patients with tongue cancer who visited Kumamoto University Hospital, Japan. A total of 53 tongue cancer patients were enrolled at the preoperative hospitalization from November 2017 to April 2019. They were diagnosed based on the histological and radiological findings, including computed tomography (CT), magnetic resonance imaging, ultrasonography, and positron emission tomography-computed tomography (PET-CT) findings. The staging of their tumors was performed according to TNM classification of the AJCC eighth edition (Amin et al., 2017). During the preoperative hospitalization, stimulated saliva samples were collected from the subjects (8.3 ± 9.4 days before cancer treatment). We instructed the subjects to chew gums and spew the whole saliva into sterile plastic tubes. After finishing the postoperative nasogastric tube feeding and starting to consume a diet orally, a similar procedure was followed to collect stimulated saliva (21.2 ± 10.9 days after cancer treatment). All post-treatment samples were collected prior to postoperative radiotherapy and chemotherapy. The samples were stored at −80°C until further analysis. After excluding 28 subjects who did not receive surgical resection (n=3), did not have both pre- and post-treatment samples (n=22), and had less than 7 teeth (n=3), 50 samples from 25 subjects were finally examined. Written informed consent was obtained from all participants. The ethics committee of Kumamoto University approved this study with the informed consent procedure (approval number 1427, 1928, and 2389).

### Quantitative PCR Analysis of Total Bacterial Density in Saliva

The collected saliva samples were subjected to quantitative PCR analysis of total bacterial density. DNA was extracted from each sample using the bead-beating method (Yamanaka et al., 2012), and quantitative PCR was performed using a QuantiFast SYBR Green PCR Kit (QIAGEN, Hilden, Germany) in QuantStudio 3 (Thermo Fisher Scientific, MA, USA) according to the manufacturer’s instructions. The primers 806F (5’-TTA GAT ACC CYG GTA GTC C-3’) and 926R (5’-CCG TCA ATT YCT TTG AGT TT-3’), with target V5 regions of the 16S ribosomal RNA (rRNA) gene, were used for the quantification of the total bacterial density (Asakawa et al., 2018).

### 16S Ribosomal RNA Gene Amplicon Sequencing of Saliva

The V1–V2 regions of 16S rRNA gene were amplified using the following primers: 8F (5′-AGA GTT TGA TYM TGG CTC AG-3′) with the Ion Torrent adapter A and the sample-specific 8-base tag sequence and 338R (5′-TGC TGC CTC CCG TAG GAG T-3′) with the Ion Torrent trP1 adapter sequence. PCR amplification, purification, and quantification of each PCR amplicon was performed as described previously (Takeshita et al., 2016). The purified PCR amplicons were pooled, and gel-purification was performed using Wizard SV Gel and PCR Clean-Up System (Promega, WI, USA). The DNA concentration was determined using a KAPA Library Quantification Kit (KAPA Biosystems, MA, USA) and the DNA was diluted for use as the template DNA in emulsion PCR. Emulsion PCR and enrichment of template-positive particles were performed using Ion PGM Template Hi-Q View OT2 Kit (Thermo Fisher Scientific) in Ion One Touch 2 System (Thermo Fisher Scientific). The enriched particle was loaded onto an Ion 318 v2 chip (Thermo Fisher Scientific) and sequencing was performed on the Ion PGM (Thermo Fisher Scientific) using Ion PGM Hi-Q View Sequencing Kit (Thermo Fisher Scientific).

### Data Analysis and Taxonomy Assignment

The quality filtering of raw sequence reads using a script written in R (version 3.6.2) was carried out. The reads were excluded from the analysis when they exhibited ≤200 bases, or had an average quality score ≤25, or did not include the correct forward, or the correct reverse primer sequence (one mismatch was allowed) or had a homopolymer of >6 nucleotides. The quality-checked reads were demultiplexed by examining the eight-base tag sequence, and then forward and reverse primer sequences were trimmed. Operational taxonomic units (OTUs) were constructed by clustering quality-checked reads, excluding singleton reads, with a minimum pairwise identity of 97% using UPARSE (Edgar, 2013) as described previously (Takeshita et al., 2016). All quality-checked reads were mapped to each OTU with ≥97% identity using UPARSE (Takeshita et al., 2016). Chimeras were identified using ChimeraSlayer and removed from analysis (Haas et al., 2011). The taxonomy of representative sequences was determined using BLAST against 889 oral bacterial 16S rRNA gene sequences (HOMD 16S rRNA RefSeq version 14.51) in the Human Oral Microbiome Database (Chen et al., 2010). Nearest-neighbor species with ≥98.5% identity was selected as candidates for each representative OTU. The taxonomy of sequences without hits were further determined using RDP classifier with a minimum support threshold of 80% (Wang et al., 2007). The number of OTUs and UniFrac distance were calculated following rarefaction to 5000 reads/sample using R. The sequence data have been deposited in DDBJ Sequence Read Archive under accession number DRA010919.

### Statistical Analysis

The bacterial characteristics of subjects pre- and post-tongue resection were compared. The total bacterial densities and diversities were compared using Wilcoxon signed-rank test for comparison of paired samples. The UniFrac metric was used to determine the dissimilarity between bacterial compositions (Lozupone and Knight, 2005). The dissimilarity between groups was evaluated using the analysis of similarities (ANOSIM) with 999 permutations based on the weighted UniFrac distance. Relative abundances of predominant genera were compared using Wilcoxon signed-rank test and obtained P-values were adjusted using a Benjamini-Hochberg false discovery rate (FDR) correction for multiple testing. The detection of discriminant bacterial species was also performed using Wilcoxon signed-rank test and FDR correction. Two-sided P < 0.05 indicated statistical significance. All statistical analyses were performed using R.

## Results

### The Characteristics of Subjects and Salivary Microbiota Sequence

A total of 25 patients with tongue cancer (16 males and 9 females, age 24–93 years old) were enrolled in the present study. The detailed characteristics of study subjects are presented in **Table 1**. Histologically, all their cancers were squamous cell carcinoma. Most of their clinical tumor (cT) stages were cT1–2 (84%) and clinical nodal (cN) stages were cN0 (92%). Distant metastasis was clinically not observed. Of the 25 subjects, 21 were administered antibiotics for biopsy (mainly amoxicillin, a beta-lactam antibiotic, n=19), and all subjects were administered antibiotics for surgery (mainly cefmetazole, a second-generation cephalosporin, n=23). Pre- and post-treatment sampling was performed on an average of 26.5 ± 9.9 and 18.6 ± 9.3 days after antibiotics exposure, respectively. Analysis of 50 stimulated saliva samples by 16S rRNA gene amplicon analysis was carried out, and 461,830 high-quality reads (9,237 ± 1,651 reads per sample) were obtained to determine their bacterial diversity and composition.

**Table 1:** The clinical characteristics of study subjects.

Clinical characteristics		
Age (years), mean ± SD		64.6 ± 18.2
Sex, n (%)	Male	16 (64.0)
	Female	9 (36.0)
Number of teeth, mean ± SD		20.6 ± 7.2
Smoking habit, n (%)	Current	8 (32.0)
	Non-current	17 (68.0)
Alcohol consumption, n (%)	Everyday	10 (40.0)
	Non-everyday	15 (60.0)
cT-stage, n (%)	1	11 (44.0)
	2	10 (40.0)
	3	4 (16.0)
cN-stage, n (%)	0	23 (92.0)
	2b	2 (8.0)
Antibiotics for biopsy, n (%)	None	3 (12.0)
	AMPC	19 (76.0)
	CDTR-PI	1 (4.0)
	CFPN-PI	1 (4.0)
Antibiotics for surgery, n (%)	CMZ	23 (92.0)
	CEZ and CTRX	1 (4.0)
	CMZ and SBT/ABPC	1 (4.0)
Reconstructive surgery, n (%)	Yes	6 (24.0)
	No	19 (76.0)

AMPC, amoxicillin; CDTR-PI, cefditoren pivoxil; CFPN-PI, cefcapene pivoxil; CMZ, cefmetazole; CEZ, cefazolin; CTRX, ceftriaxone; SBT/ABPC, sulbactam/ampicillin; cT-stage, clinical tumor stage; cN-stage, clinical nodal stage; SD, standard deviation.

### Shift of Total Bacterial Density in Saliva Following the Surgical Resection

A quantitative PCR analysis was performed to evaluate the effect of the surgical resection on the total bacterial density of salivary microbiota. As shown in **Table 2**, there was no significant difference in pre- and post-treatment samples (P = 0.35).

**Table 2:** Bacterial density and diversity in pre- and post-treatment samples.

	Pre-treatment (n=25)	Post-treatment (n=25)	P value
Bacterial density (log copies/ml), mean ± SD	9.85 ± 0.41	9.96 ± 0.33	0.35
Bacterial diversity			
Number of OTU, mean ± SD	147.5 ± 31.1	132.6 ± 22.9	0.015
Shannon index, mean ± SD	3.4 ± 0.35	3.4 ± 0.30	0.58

SD, standard deviation.

### Shift of Bacterial Diversity and Bacterial Composition in Saliva Following the Surgical Resection

**Figure 1:**
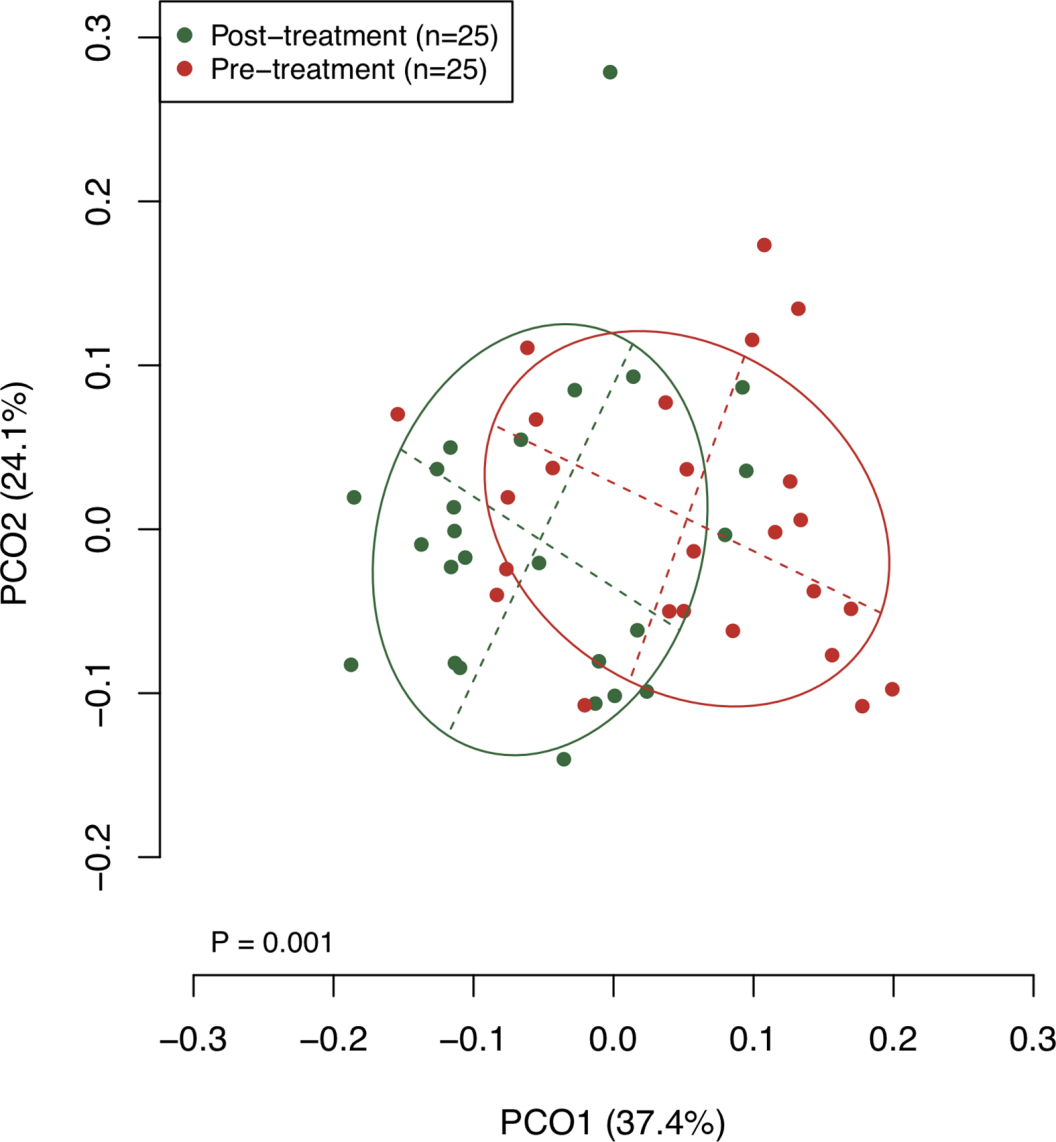
A principal coordinate analysis (PCoA) based on weighted UniFrac distance.**** The bacterial composition of pre- and post-treatment samples are depicted using different colors. These two components explain the 61.5% variance. The intersection of the broken lines indicates the center of gravity for each group. The ellipse covers 67% of the samples belonging to each group.

The collected saliva samples were examined using 16S rRNA gene sequencing to evaluate the effect of the surgical resection on the bacterial balance of salivary microbiota. The post-treatment saliva exhibited significantly lower bacterial diversity than the pre-treatment saliva according to observed number of OTUs (P = 0.01, **Table 2**). **Figure 1** presents a principal coordinate analysis (PCoA) plot based on the weighted UniFrac distances. According to ANOSIM, there was a significant difference in bacterial composition of salivary microbiota pre- and post-surgery (P = 0.001). On confirmation of the bacterial composition of their salivary microbiota at genus level, 18 predominant genera with ≥1% of the relative abundance accounted for 93.4 ± 4.4 and 93.3 ± 5.3% in pre- and post-treatment samples, respectively. Among them, post-surgical resection, *Streptococcus*, *Prevotella*, *Gemella*, and *Leptotrichia* were significantly decreased while, *Neisseria*, *Fusobacterium*, and *Lautropia* were significantly increased.

### Bacterial Species Showing Drastic Compositional Shift Following the Surgical Resection

**Figure 2:**
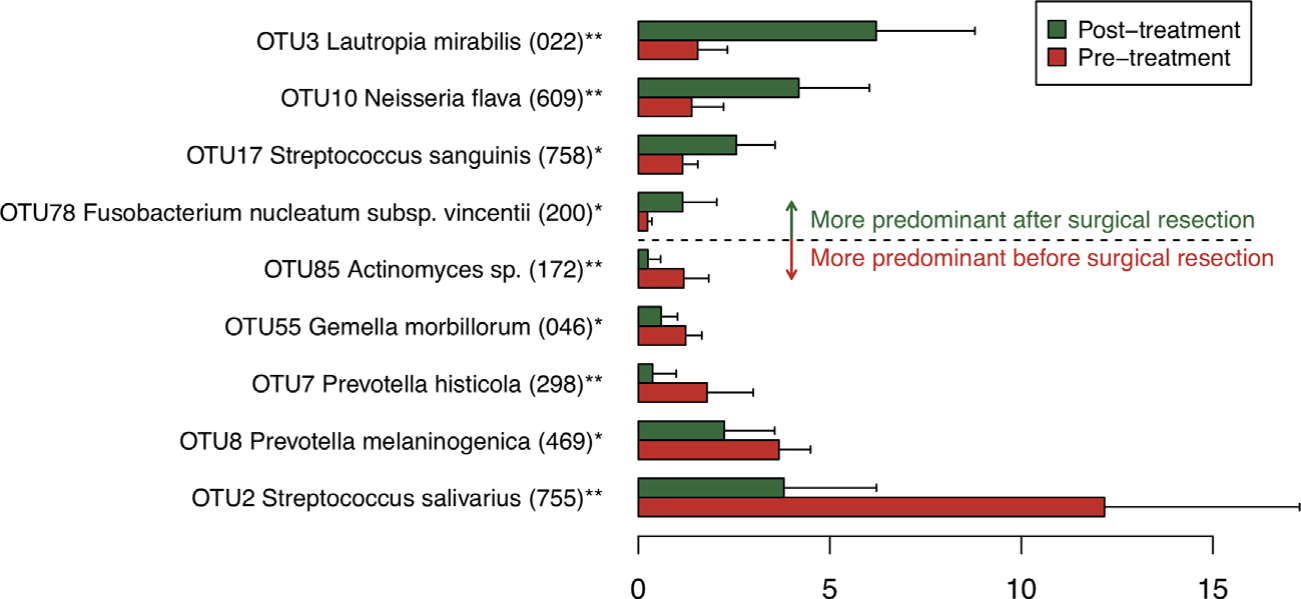
Bacterial species corresponding to the differentially abundant operational taxonomic units (OTUs) between pre- and post-treatment samples. Bar plots show mean relative abundances of differentially abundant OTUs. Only nine OTUs with ≥1% of the relative abundance and significant difference were shown. The bar plots of pre- and post-treatment samples are depicted using different colors. Error bars indicate 95% confidence intervals. Oral taxon IDs are given in parentheses following bacterial names. **P < 0.01. *P < 0.05.

To find bacterial species that were drastically increased or decreased following the tongue resection, discriminant OTUs in pre- and post-treatment samples were identified using the Wilcoxon signed-rank test. Among predominant OTUs with ≥1% of the relative abundance, the analysis revealed nine OTUs were differentially abundant in pre- and post-treatment samples (**Figure 2**). Following the surgical resection, OTUs corresponding to *Streptococcus* *salivarius* HOT-755, *Prevotella* *melaninogenica* HOT-469, *Prevotella* *histicola* HOT-298, *Gemella morbillorum* HOT-046, and *Actinomyces* species HOT172 were significantly decreased, whereas OTUs corresponding to *Lautropia* *mirabilis* HOT-022, *Neisseria* *flava* HOT-609, *Streptococcus* *sanguinis* HOT-758, and *Fusobacterium nucleatum* HOT-200 were significantly increased.

## Discussion

The present study demonstrated that following surgical resection of tongue cancer, the bacterial diversity decreased, and the bacterial balance of predominant bacteria in salivary microbiota shifted. In post-treatment samples, *L. mirabilis*, *N. flava*, *S. sanguinis*, and *F. nucleatum* were significantly increased compared to that in the pretreatment samples, and they were all oral indigenous bacteria. This result suggests that surgical resection causes a balance shift of salivary microbiota, but not drastic and terrible oral dysbiosis, such as overgrowth of non-indigenous pathogenic bacteria. On the other hand, *F. nucleatum*, a periodontal pathogen, increased in salivary microbiota following tongue resection. *F. nucleatum* generally inhabits subgingival plaque and has periodontopathogenic properties, such as activation of inflammatory cytokines that lead to periodontal attachment and tissue damage (Baqui et al., 1998). In addition, *F. nucleatum* is considered an opportunistic pathogen implicated in the carcinogenesis of oral cancer, not only periodontitis (Gholizadeh et al., 2016). Interestingly, although it generally inhabits the oral cavity, several studies suggest its association with gastrointestinal diseases, such as inflammatory bowel disease and colorectal cancer by inducing inflammation and downregulating host immunity (Strauss et al., 2011; Mima et al., 2015; Nosho et al., 2016; Wu et al., 2019). In fact, *F. nucleatum* is frequently and abundantly detected in colorectal tissue from patients with these diseases (Strauss et al., 2011; Mima et al., 2015; Nosho et al., 2016; Yachida et al., 2019). As swallowing function often declines after tongue resection, assessment of salivary microbiota in postoperative patients might help in preventing the development of associated gastrointestinal diseases as well as oral diseases.

In the discriminant analysis, the bacterial species, *L. mirabilis*, *N. flava*, *S. sanguinis*, and *F. nucleatum*, which mainly inhabit dental plaque, were increased after resection of the tongue (Takeshita et al., 2015; Kageyama et al., 2017; Ihara et al., 2019). On the other hand, *S. salivarius*, *P. melaninogenica*, *P. histicola*, and *Actinomyces* species, known as predominant species in tongue dorsum, were identified as decreasing species (Kageyama et al., 2017; Asakawa et al., 2018). Although the preferred habitat of *G. morbillorum* remains unclear, all other discriminant bacteria demonstrated a common tendency: an increase in dental plaque bacteria and a decrease in tongue bacteria. These results suggest that oral environment post-surgical resection alters by reduction in surface area of tongue, and dental plaque-derived bacteria become dominant in salivary microbiota. These findings are in accordance to the previously proposed concept of tongue microbiota being a major source of salivary microbiota.

Of the 25 subjects who underwent surgical resection of tongue cancer, reconstructive surgery was performed in six subjects (4 men and 2 women), and their surgical defects were reconstructed using a cervical island skin flap (n=3), pectoralis major myocutaneous flap (n=2), and skin graft (n=1). The tongue dorsum is lined by stratified squamous epithelium with numerous tongue papillae, which provide an anaerobic environment for harboring diverse anaerobic bacteria. The complex papillary structure also retains blood serum components, infusion from the gingival crevice, epithelial cells, and food residue on the tongue surface as tongue coating and provide a nutrient-rich environment for tongue bacteria. On the other hand, the skin graft and flap are lined by the stratified squamous epithelium with thick keratinized layer, and the surface structure is relatively smooth. Thus, it is reasonable to assume that this difference changes the bacterial species on the tongue surface and consequently changes the bacterial composition of the salivary microbiota. However, there was no significant difference in the bacterial composition of post-treatment saliva with or without reconstructive surgery (data not shown). In this study, saliva samples were collected at 21.5 ± 10.5 days post-surgery to identify the effect of tongue resection. The formation of tongue coating and tongue microbiota in the long term may differ on the original tongue surface or the grafted surface, and the results would be fundamental for the development of a novel approach to control the virulence of oral microbiota. Further studies are required to elucidate the long-term effects of the skin graft and flap on salivary and tongue microbiota.

This study has several potential limitations. Firstly, most subjects of the present study were administered antibiotics for biopsy and surgery prior to pre-treatment and post-treatment sampling, respectively. Of the 25 subjects, 21 subjects were administered antibiotics for biopsy and all subjects were administered antibiotics for surgery. However, previous report indicated that bacterial composition of the salivary microbiota was stable against antibiotic treatment compared to feces microbiota, and impacts of antibiotics including ciprofloxacin, amoxicillin, and minocycline on the salivary microbiota composition were lost by 1 week to 1 month post-exposure (Zaura et al., 2015). Another report also suggested the impact of amoxicillin on salivary bacteria peaked out 4 h after exposure (Larsson Wexell et al., 2016). In this study, the pre- and post-treatment sampling were performed on an average of 26.5 ± 9.9 and 18.6 ± 9.3 days after antibiotics exposure, respectively. Although most samples were collected within a month after administration of antibiotics, it was unlikely that the effect of antibiotics remained robust, especially for a selective increase of dental plaque-derived bacteria. Secondly, although oral conditions such as number of teeth, periodontal conditions, and dental caries status affect the bacterial diversity and composition of salivary microbiota, however, the data about dental examination including periodontal pocket depth, bleeding on probing, decayed teeth, and oral hygiene status was not collected, except number of teeth from panoramic radiographs. This limited the thorough understanding of the effect of tongue resection on the salivary microbiota. However, considering dental caries and periodontitis gradually progress, there was probably no drastic change in periodontal pocket depth and dental caries status between samplings. It is also speculated that oral hygiene status was unchanged because of the stable bacterial density in saliva before and after the surgery (**Table 2**). Lastly, the sample size of the present study, especially those who underwent reconstructive surgery, was small. Although smoking is known as a lifestyle factor affecting the oral microbiota (Takeshita et al., 2016), a significant difference by smoking was not observed in the bacterial composition of pretreatment samples, post-treatment samples, and a bacterial composition shift following the surgery. Similar results were obtained for alcohol consumption. In addition, although *F. nucleatum* increased in the salivary microbiota following tongue resection, the shift in the overall virulence of salivary microbiota remains unclear. Further studies with larger sample sizes are required to identify the effects of surgical resection and reconstructive surgery on the bacterial composition and the virulence of oral microbiota as well as the effects of lifestyle factors on oral microbiota in the perioperative period.

In conclusion, surgical resection of tongue cancer causes a shift in the bacterial diversity and composition of salivary microbiota characterized by an increase in bacterial species derived from dental plaque. In particular, *F. nucleatum* is a periodontal pathogen and is suspected to be associated with oral and gastrointestinal diseases. These results might suggest the necessity of more careful and frequent postoperative oral care after surgical resection of tongue cancer.

## Data Availability Statement

The datasets presented in this study can be found in online repositories. The names of the repository/repositories and accession number(s) can be found at: https://www.ncbi.nlm.nih.gov/sra/, accession no: DRA010919.

## Ethics Statement

The studies involving human participants were reviewed and approved by The ethics committee of Kumamoto University. The patients/participants provided their written informed consent to participate in this study.

## Author Contributions

SK wrote the first draft of the manuscript. SK, YN, RY, AH, TT, YY, and HN critically revised the manuscript. YN, RY, AH, and HN collected the clinical data and sample. YN, JM, and MA performed the molecular analysis. SK, JM, MA, and TT performed the bioinformatics, and statistical analysis. SK, YN, AH, TT, YY, and HN contributed to the conception and design of the study. All authors contributed to the article and approved the submitted version.

## Funding

This work was supported by JSPS KAKENHI Grant Numbers JP20K18808, JP20H03901, JP19K22722, JP19H03863, JP18H03005, and JP18K09727.

## Conflict of Interest

The authors declare that the research was conducted in the absence of any commercial or financial relationships that could be construed as a potential conflict of interest.
